# Wireless Ultrasound Surgical System with Enhanced Power and Amplitude Performances

**DOI:** 10.3390/s20154165

**Published:** 2020-07-27

**Authors:** Jungsuk Kim, Kiheum You, Sun-Ho Choe, Hojong Choi

**Affiliations:** 1 Department of Biomedical Engineering, Gachon University, 534-2, Hambakmoe-ro, Incheon 21936, Korea; jungsuk@bme.gachon.ac.kr; 2 Department of Medical IT Convergence Engineering, Kumoh National Institute of Technology, 350-27 Gumi-daero, Gumi 39253, Korea; rlgma12@kumoh.ac.kr; 3 R&D Center, Metabiomed Corporation, 215 Osongsaenmyeong1-ro, Chenongu 28161, Korea; sognatore@metabiogw.bizmeka.com

**Keywords:** wireless ultrasound surgical system, ultrasound transducer, battery module

## Abstract

A wireless ultrasound surgical system (WUSS) with battery modules requires efficient power consumption with appropriate cutting effects during surgical operations. Effective cutting performances of the ultrasound transducer (UT) should be produced for ultrasound surgical knives for effective hemostasis performance and efficient dissection time. Therefore, we implemented a custom-made UT with piezoelectric material and re-poling process, which is applied to enhance the battery power consumption and output amplitude performances of the WUSS. After the re-poling process of the UT, the quality factor increased from 1231.1 to 2418 to minimize the unwanted heat generation. To support this UT, we also developed a custom-made generator with a transformer and developed 2nd harmonic termination circuit, control microcontroller with an advanced reduced instruction set computer machine (ARM) controller, and battery management system modules to produce effective WUSS performances. The generator with a matching circuit in the WUSS showed a peak-to-peak output voltage and current amplitude of 166 V and 1.12 A, respectively, at the resonant frequency. The performance with non-contact optical vibrators was also measured. In the experimental data, the developed WUSS reduced power consumption by 3.6% and increased the amplitude by 20% compared to those of the commercial WUSS. Therefore, the improved WUSS performances could be beneficial for hemostatic performance and dissection time during surgical operation because of the developed UT with a piezoelectric material and re-poling process.

## 1. Introduction

Ultrasound transducers (UTs) have been widely used in ultrasound identification, sound navigation ranging, non-destructive testing, cleaning, smartphone touchpads, and medical applications [1,2,3,4,5,6,7]. The medical applications include imaging, therapeutics, and surgical systems [8,9,10]. Ultrasound surgical systems are used for coagulation, incision, and hemostasis for animal or human tissues [11].

The global market of the ultrasound surgical system (USS) is increasing by an average of approximately 6.5% annually owing to the increased demand for minimally invasive surgery [12]. The energy device in the surgical system market is also expected to grow at an annual average of approximately 10.8%, reaching $ 5.2 billion by 2025 [12]. Because of increased minimally invasive and laparoscopic surgeries, the USSs for soft tissues are increasing [13]. Recently, Covidien Corporation has developed a wireless-type USS with unique battery power modules [14]. The operating frequency of the wireless-type USS is 55.5 kHz for coagulation, incision, and hemostasis of blood vessels [14].

The wireless ultrasound surgical instrument (WUSS) is composed of a hand-piece, hand, and generator instrument with a battery module, including charger parts [15]. In the hand-piece, acoustic vibration at a specific frequency is generated in the UT modules [13]. In the hand instrument, customers or physicians can control the coagulation, incision, and hemostasis operations [16]. In the generator instruments, electrical powers are generated to trigger the UT [17,18,19]. The battery with a charger provides power to the generator instruments. For USSs, a bolt-clamped-Langevin transducer, a type of UT, is used for high power applications, such as ultrasound surgical systems [20]. The UT used for cutting soft tissues in surgical operation have been widely developed around 1990s, however, currently developed UT is still difficult to dissect large size soft tissues until now [21]. Therefore, improved vibration amplitude of UT is helpful to dissect and coagulate the soft tissues.

A few researchers have been recently developed to improve the vibration amplitude and output frequency performances of the UTs or instruments for surgical purposes. In 2005, the ultrasonically activated scalpel was developed to improve the bending mechanism. The measured frequency and vibration amplitude of the device is around 47 kHz and 18 µm [22]. Developed bended UT is useful to provide stable vibration amplitudes even with 50° bended position. In 2012, silicon micromachined UT with PZT material was developed to improve power transfer capability during cutting operation. The frequency ranges of the developed UT are from 50 to 100 kHz with 36 µm displacements [23]. In 2017, the microstructures of the surgical tip with Ti-6AL-4V material was fabricated to improve acoustic powers of the UT [24]. In 2019, the d_31_-mode supported ceramic piezoelectric transducer was developed to provide reliable vessel cutting capability for minimally invasive surgical robot operation [25]. The cutting operation time was reduced to be around 10 sec for 330 burst pressure [25]. The ultrasonic scalpel with Ti6A114V alloys was developed with resonant frequency of 55.06 kHz and vibration amplitude of 21.48 µm. In 2020, a UT for ultrasonic scalpel with 29.3 kHz resonant frequency and 40 µm vibration amplitude with 16.6 sec dissection time was recently developed [26]. Current research direction of the developed UT and instrument for soft tissue surgical operation is to enhance the output amplitude and driving frequency performances [21].

Currently, most developed USSs utilize UTs that contain Pb[Zr_x_Ti_1−x_]O_3_ (PZT)-based piezoelectric materials [14]. The UT materials for USSs are typically PZT-4 (Navy type I) or PZT-8 type (Navy type III) piezoelectric materials [27]. PZT-4 and PZT-8 piezoelectric materials have a low dielectric loss but a high mechanical factor; therefore, they are also used for ultrasonic welding or cleaning applications [27,28]. However, in this study, we used Mn-doped PIN-Pb(Mg_1/3_Nb_2/3_)O_3_-PbZrO_3_-PbTiO_3_) (Mn: PIN-PMN-PZT) piezoelectric material and newly developed re-poling process for the WUSS-based on our best knowledge.

In the previous wire ultrasound surgical system, PMN-PZT single crystal piezoelectric material was used [29]. In the previous work, we used compressed forces using the developed alignment system to obtain more accurate frequency control of the system. However, the wireless ultrasound surgical system has required higher output voltage and vibration amplitude to enhance the hemostasis and incision time since portable battery life would limit those performances. Therefore, in the WUSS, we used piezoelectric material, which is Mn: PIN-PMN-PZT single crystal piezoelectric material.

This piezoelectric material was also fabricated with newly developed re-poling process to improve the quality factor of the UT. Therefore, this material could provide higher quality factor such that it could provide higher power with same sizes because higher quality factor of the piezoelectric material could improve the hemostasis performance [30]. Compared with PZT-based piezoelectric materials, Mn: PIN-PMN-PZT piezoelectric materials have higher output power per size and volume, and higher power can be expected through re-poling process; thus, it is beneficial for the WUSS performances.

Compared to previous work, we also used second harmonic termination circuit with transformer in the designed transimpedance amplifier. It is useful to reduce harmonic distortion of the applied ultrasound signals partially caused by battery management system modules [31]. In addition, the spring-type pogo pin was utilized to enhance the durability of fixing and attachment to battery management system terminal block which is useful to check the maximum/minimum/open status more accurately.

It can also be used to manufacture a low-weight product such as the WUSS for physicians who require low weight during an extended surgical operation [8]. However, the Mn: PIN-PMN-PZT piezoelectric material has limited maximum voltages compared to those of PZT piezoelectric materials. Therefore, UTs that contain Mn: PIN-PMN-PZT piezoelectric material should be efficiently designed with respect to the instrument side. An appropriate design of the UT with the instrument could be challenging to boost the performances of the WUSS.

The UT designed for USS applications should generate maximum displacement at the distal end, where the waveguide is connected when considering the wavelength of each piezoelectric material. The speed of sound in the piezoelectric material is [31,32,33]

(1) $$c=\sqrt{\frac{E}{\rho }},$$

where *E* is Young’s modulus, and *ρ* is the density of the material.

The resonant frequency (*f*) in the first longitudinal mode is expressed as follows [34,35].

(2) $$f=\frac{1}{2l}\sqrt{\frac{E}{\rho }}$$

Therefore, the length of the piezoelectric material (*l*) can be expressed by

(3) $$l=\frac{1}{2f}\sqrt{\frac{E}{\rho }}=\frac{c}{2f}=\frac{\lambda }{2}$$

where *λ* is the wavelength of the piezoelectric material.

Section 2 describes the design and fabrication of the UT, hand-piece, and generator instruments. Section 3 describes the implementation and measurement results of the UT in the hand-piece and generator instruments with the battery charger. The measured performances of the WUSS using non-contact optical vibrator equipment are also discussed.

## 2. Materials and Methods

**Figure 1:**
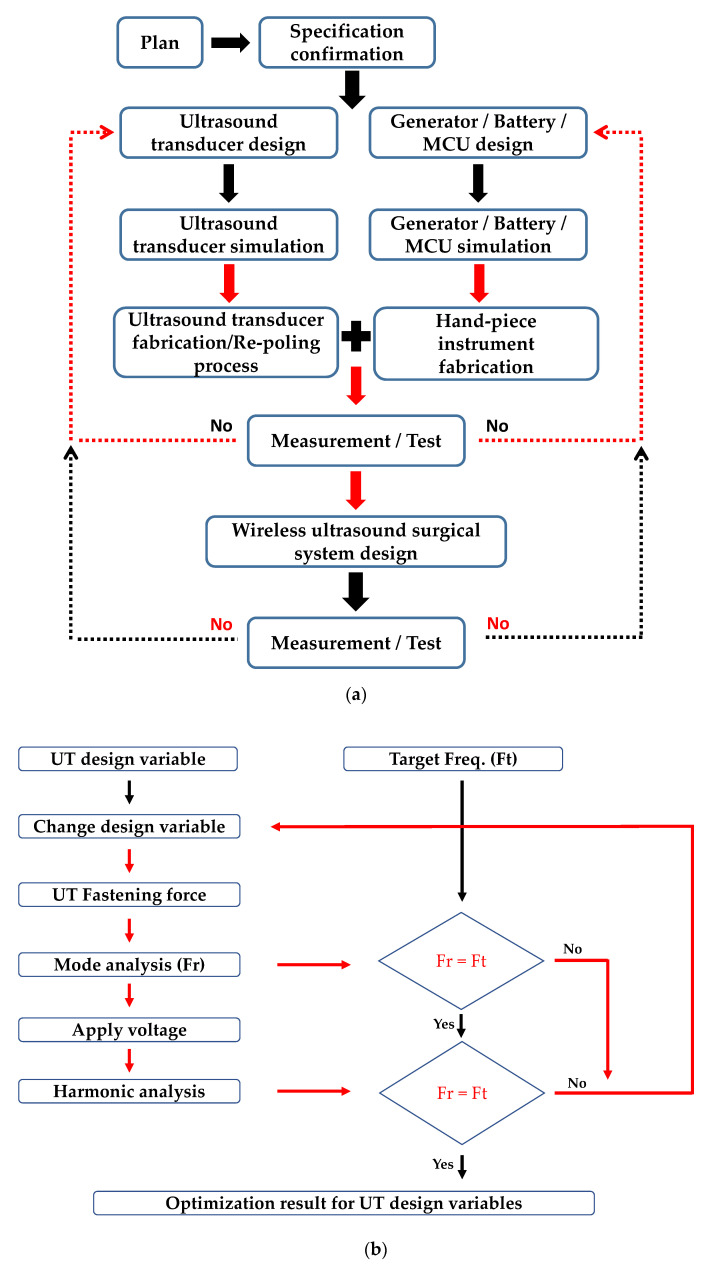

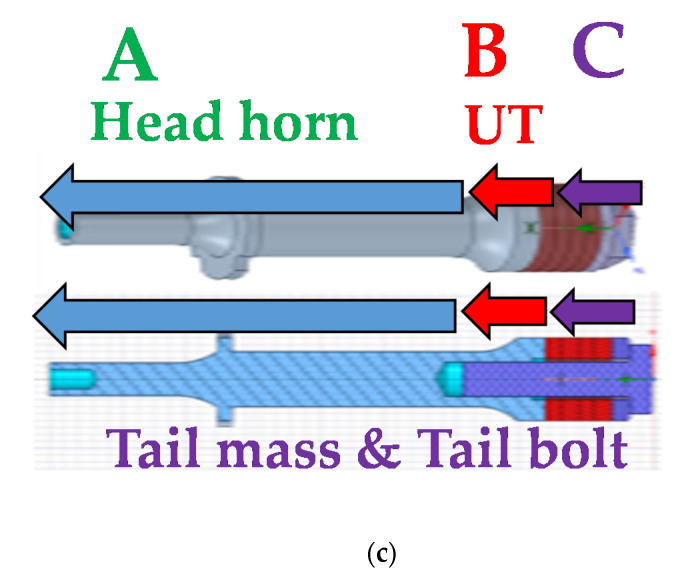
(**a**) Schematic diagram to describe the overall methodology for the developed WUSS. (**b**) Optimization procedure of the UT and (**c**) three-dimensional (3D) model for the UT with head horn, and tail mass and bolt design.

Figure 1a shows the overall methodology for the developed WUSS. First of all, the specification of overall WUSS was determined. The UT needs to be designed with simulation tool and then, fabricated the UT with aluminum cases. With re-poling process, the UT performances could be stabilized. The generator, battery, and microcontroller unit (MCU) were designed and they are integrated with hand-piece instrument. After test and measurement of the hand-piece instrument, the UT, and electronics, wireless ultrasound surgical system could be implemented accordingly.

### 2.1. Ultrasound Transducer Design

The developed UT is composed of a piezoelectric single crystal with a head horn, tail mass, tail bolt, electrodes, and insulation tube. The head horn was used to amplify the ultrasonic vibration in the thickness mode at a constant cross-sectional ratio [36]. The tail mass was used to absorb and reflect the wavelength in the rear direction among the acoustic vibration generated in the front and rear directions and the cooldown heat generated in the UT; therefore, a high strength Ti-6Al-4V material was used [37]. The electrodes were used to apply electrical signals to the piezoelectric materials. BeCu material with high electrical conductivity and elasticity was used [38]. To prevent short circuits between the piezoelectric body and electrodes, PFA tube material, which is a fluorine resin with a low coefficient of friction, was used for the insulation tube, which was inserted into the threaded bolt and screw [39].

A single crystal piezoelectric material (PIN-PMN-PZT), which is used in ultrasonic transducers for imaging machines, exhibits a lower electric field under stress than that of a hard polycrystalline piezoelectric material (PZT-8) [40,41]. Thus, the piezoelectric material performances deteriorate when high power is driven to the system. The low mechanical power makes it difficult to apply these materials to high-power vibration devices in ultrasonic surgical systems [31,42]. Because of the excellent piezoelectric properties and stability, Mn was doped into the developed PIN-PMN-PZT-based single crystal piezoelectric material to improve the stability of the high output driving characteristics, thus possibly enhancing the UT performances.

According to the single-crystal piezoelectric property measurement results of the Mn: PIN-PMN-PZT material, the quality (Q_m_) factor, which corresponds to the stability of the driving characteristics regarding heat generation, is 1231.19. The constant electric field (E_c_) characteristic measurement under a no-load condition is 4.3 kV/cm, allowing a 2.5 mm thickness single crystal ring to drive the high voltage within 1 kV.

The UT, which supports high power operation, was designed to increase the operation time of the battery in the WUSS. To design this UT, multi-physics modeling software (COMSOL Inc., Stockholm, Sweden) was used to optimize the maximum performances at the resonant frequency for the design parameters [43]. Figure 1b shows the design process of UT. As shown in Figure 1c, the optimal shape with head horn (A), UT (B), and tail mass and bolt (C) was designed by repeating the process of defining the shape, properties, optimum condition, numerical analysis, and results.

### 2.2. Ultrasound Transducer Simulation

**Figure 2:**
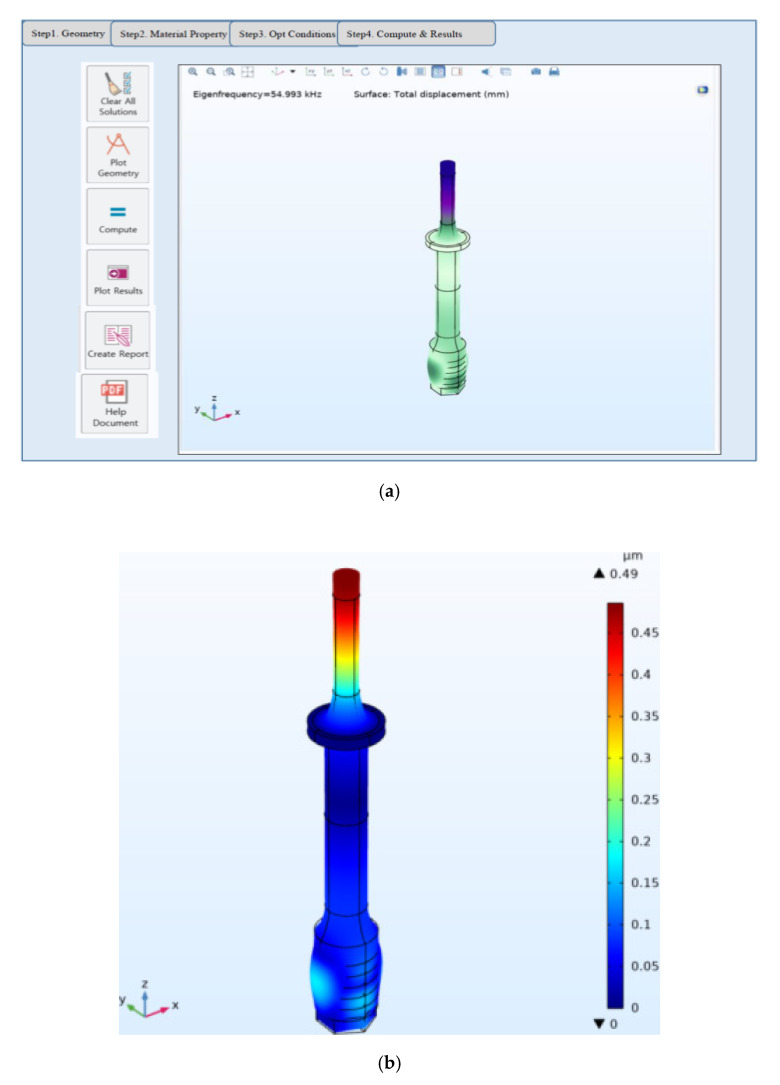

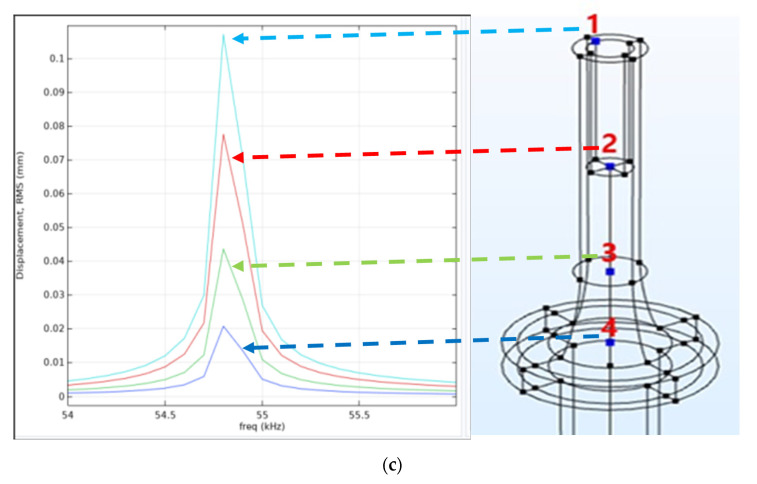
(**a**) Optimizer design setup, (**b**) modal analysis result, and (**c**) harmonic analysis result for the UT.

Figure 2a shows the optimizer design setup for the designed UT. To reduce the weight, the ring diameter was reduced to 12 mm, and the subordinated metal parts were set as a fixed variable. For accurate shape design, the measured physical properties of the metal and piezoelectric materials were applied. Afterward, the optimum shape was designed by repeating the process of defining the shape setting optimum conditions and numerical analysis. As shown in Figure 2b, the modal analysis confirmed that the first longitudinal vibration mode is similar to the target frequency of 55.5 kHz, and maximum displacement occurs at the end of the head horn. Figure 2c shows the harmonic analysis result of the UT. In the harmonic analysis results, based on the fixed end of node point 4, the displacement is higher when closer to head horn endpoint 1; however, it may be different from the actual state because it is the steady-state analysis result. Based on the results of the modal and harmonic response analyses, a single-element low-power UT was manufactured.

Table 1 lists the metal material properties for the UT. Optimal shape dimensions satisfying the design parameters were derived to minimize the trial and error of fabrication by selecting a suitable shape. The head horn was made of AL7075 material [44]. The tail mass and tail bolt were made of high strength Ti-6Al-4V [45].

### 2.3. Ultrasound Transducer Fabrication and Re-Poling Process

**Figure 3:**
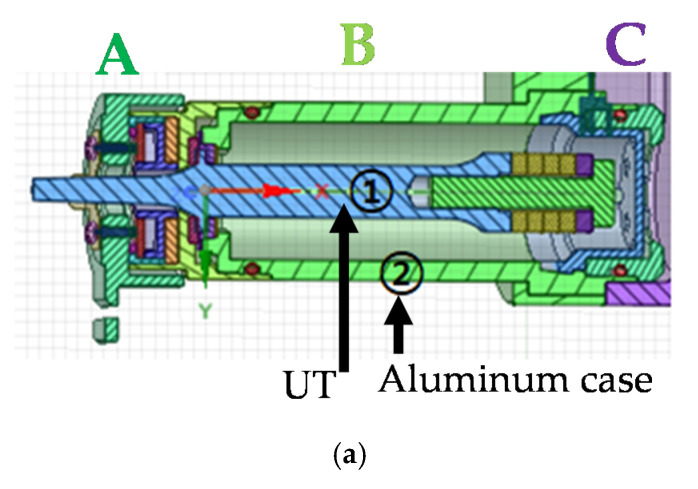

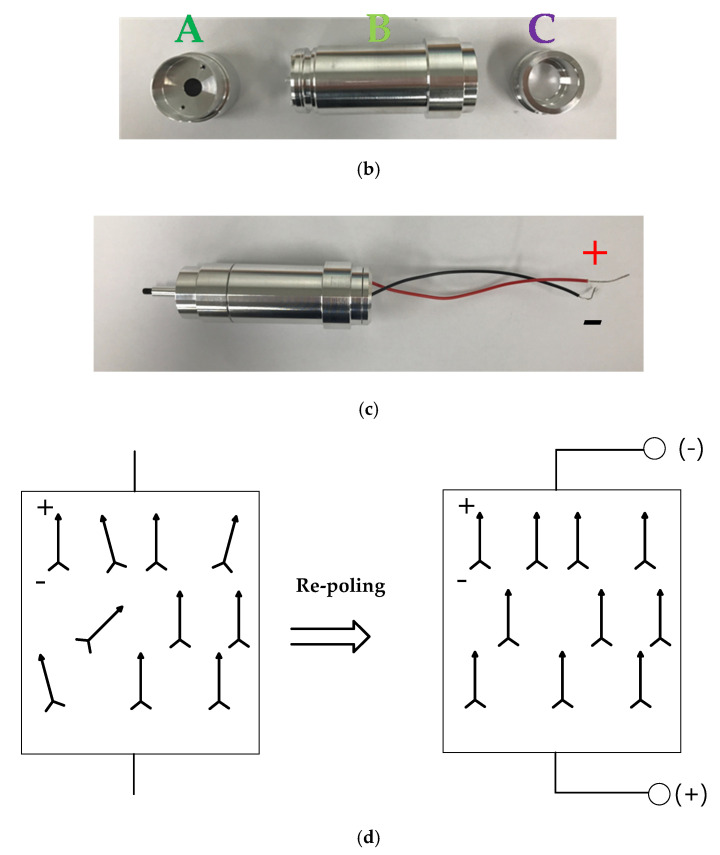
(**a**) Hand-piece model, (**b**) aluminum case, (**c**) assembled hand-piece instrument, and (**d**) re-poling process method.

The hand-piece instrument is composed of the UT (①) with an aluminum case (②), as shown in Figure 3a. In the hand-piece instrument, there are three body parts such as hand instrument body connector (A), case body (B), and cable body connector (C). The case contains three stages for the UT, as shown in Figure 3b, and it was composed of aluminum alloy for durability and weight. Figure 3c shows the assembled hand-piece instrument.

We utilized the re-poling process for the developed UT fabricated with Mn: PIN-PMN-PZT material to improve the UT quality factor, which could reduce unwanted power consumption in the WUSS battery modules. The re-poling process of the piezoelectric transducer utilizes multiple poling processes [27]. It could be effective for our developed UT with Mn: PIN-PMN-PZT material to increase the quality factor. As shown in Figure 3d, some of the internal electric dipoles are not aligned owing to aging effects on the piezoelectric material after fabricating the UT. After integrating the unwanted excessive forces on the piezoelectric material, a physical influence on the piezoelectric element, such as increasing the range between the resonant and anti-resonant frequencies, was generated. Therefore, the re-poling process method was utilized to apply a direct current (DC) power to a previously determined piezoelectric material again under the same environment as that during the fabrication of the piezoelectric material. The operating environment for the single crystal material was the Currie temperature. Therefore, the internal electric dipoles could be aligned again. The ranges of the resonant and anti-resonant frequencies of the piezoelectric transducer after the re-poling process could be reduced again, thus improving the UT quality factor.

Commercial polycrystalline UT should use a backing process for short-term aging characteristic stabilization by heating the material at 100 °C for approximately 10 min. However, a backing process is difficult to apply to our developed piezoelectric material in the UT owing to the relatively low temperature. Therefore, we stabilized the performances of the UT with a re-poling process that re-arranges polarization at 80 °C instead of a backing process. Thus, the aging characteristic of the material is stabilized within 5 h, while the aging characteristic of the single crystal material without the re-poling process lasts approximately two weeks or longer. Therefore, the UT performances could be stabilized after integrating the system hardware, such as the hand-piece and generator instruments. The stabilized characteristics of the UT could enable more accurate matching and operating with the generator instrument. Without this re-poling process, the integration between the hand-piece and battery power module could cause unstable operation of the hand-piece, reducing the output efficiency of the WUSS. As a result of the re-poling process of the UT, the quality factor is increased so it is helpful to enhance the WUSS power performances.

### 2.4. Generator and MCU Module Design

**Figure 4:**
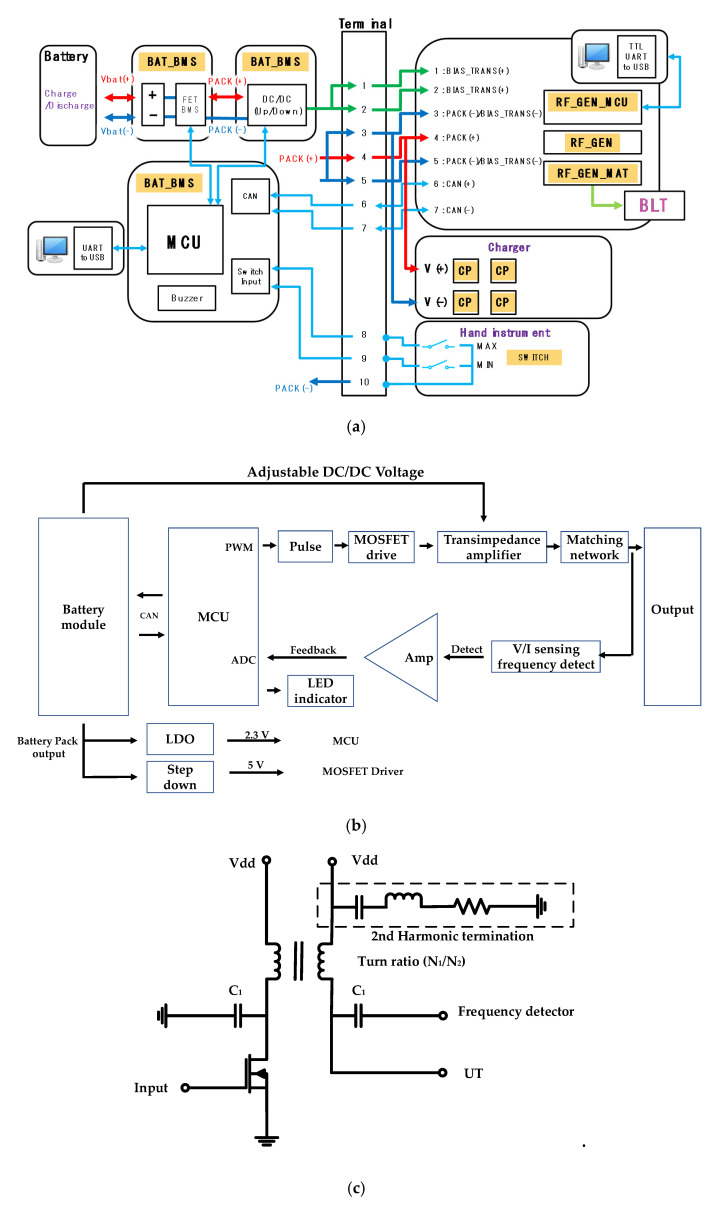

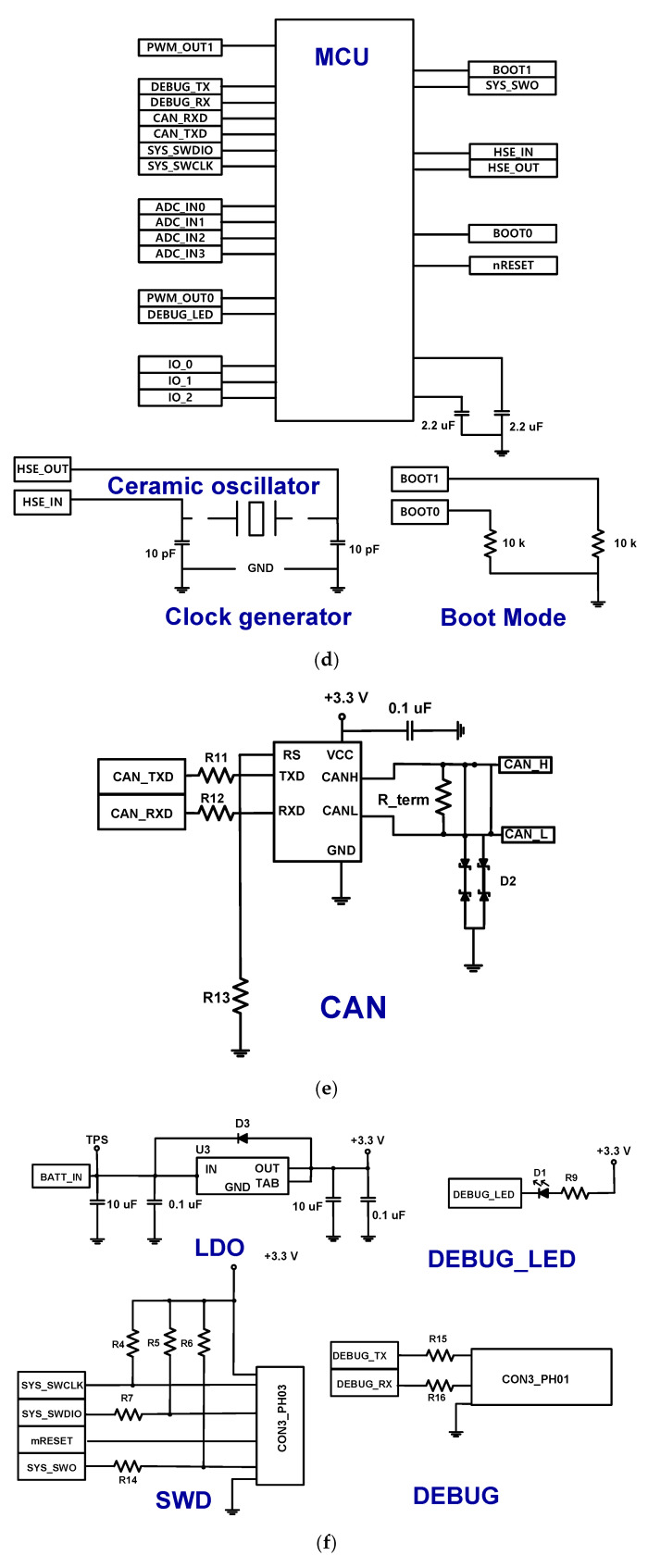
(**a**) Configuration of the wireless ultrasound surgical system (WUSS), (**b**) block diagram of the generator instrument, and (**c**) designed transimpedance amplifier circuit with transformer and harmonic termination parts. Circuit diagram of the (**d**) main microcontroller unit (MCU), (**e**) controller area network (CAN) communication driver, and (**f**) low-drop out (LDO), serial wire debug (SWD), and debugging interfaces.

The WUSS (Figure 4a) is composed of the hand-piece, generator, and battery with charger instruments. The batter instrument consists of the battery management system (BAT_BMS), DC/DC converter (DC/DC (Up/Down)), MCU unit, and high/low (H/I) switch. In the battery modules, two 3.7 V Li-ion batteries with 2.8 A maximum current including charge and discharge protection circuits were used. From the BAT_BMS(Vbat(+)/(−)), the battery management system (BAT_BMS) provides the input of battery DC/DC converter (DC/DC). The battery DC/DC converter provides two inputs of the transformer used in the trans-impedance amplifier (BIAS_TRANS(+)/(−)). Controller area network (CAN) communication ports (CAN(+)/(−)) were performed between the battery module and MCU.

The generator instrument consists of the pulse generator (RF_GEN), matching network (RF_GEN_MAT), and MCU controller circuits (RF_GEN_MCU). In the generator instrument, the pulse formed by the MCU (RF_GEN_MCU) is used to drive the pulse generator (RF_GEN) circuit through the matching circuit (RF_GEN_MAT) to excite the UT. The DC voltages are applied to the transformer (BIAS_TRANS(+)/(−)). The positive battery voltage (PACK(+)) and ground battery voltage (PACK(−)) are used. Universal asynchronous receiver/transmitter (UART) communication was used to provide the input through USB ports from the computer. In the hand instrument, there are two switches for maximum and minimum allowable current (MAX/MIN) for ultrasound outputs. The MCU controls the current through internal switches (Switch Input) and also controls the buzzer (Buzzer) with appropriate sound pressure to generate the alarm. In the charger instrument, there are charge pack (CP) with +/− DC voltages (V(+)/(−)) for several batteries.

As shown in Figure 4b, the pulse width modulation (PWM) chip provides the input of the pulse forming circuit (Pulse) through the metal-oxide-semiconductor field-effect transistor (MOSFET) driver with two different 180° phase and 50% duty cycle. The received input was amplified through transimpedance amplifier via matching network. The generator output signal of the trans-impedance amplifier and matching network was detected via V/I sensing circuits. The detected input signals were amplified through operational amplifier (Amp) and transferred to the analog-to-digital converter (ADC) in the MCU as an input. The battery module output could adjust the DC voltages of transimpedance amplifier. The battery module output also provides the input to the low-dropout (LDO) and step-down integrated circuit (IC) with 2.3 V and 5V DC. The LDO and step-down IC provide the required voltages to each MCU and the MOSFET driver in the transimpedance amplifier. Therefore, each necessary alarm signals can be generated with detected voltage, current, and frequency values.

Figure 4c shows the designed trans-impedance amplifier with 2nd harmonic termination circuits. The trans-impedance amplifier is a kind of common-source amplifier type with transformer and 2nd harmonic termination circuit. The 2nd harmonic terms typically deteriorate the acoustic signal waveform of the UTs; therefore, the resistance, capacitance, and inductance values in the 2nd harmonic termination are determined [46,47]. Even, highly distorted signals could generate the acoustic signals with inaccurate frequency components from WUSS; therefore, this could possibly reduce the WUSS performance. In the trans-impedance amplifier, the turn ratio of the transformer is 0.06, and the capacitance value used to connect the frequency detector which has 480 nF (C_1_). Therefore, transformer and 2nd harmonic termination circuit could be useful to obtain clear acoustic signal waveform for WUSS.

Figure 4d,e show the circuit diagram of the main MCU and CAN communication driver used to communicate with the battery management system (BMS ) board. Figure 4f shows the circuit diagram of the LDO, serial wire debug (SWD), and debugging interfaces. The control MCU was designed for appropriate performance of the WUSS. The advanced reduced instruction set computer machine (ARM) controller (STM32F405R, STMicroelectronics) was used to control the output controller using external signals.

In Figure 4d, each pin was connected to general-purpose input/output (GPIO) pin. The boot loader needs to be placed to re-program system memory in the boot mode by using ports (DEBUG_TX, DEBUG_RX, DEGU_LED, CAN_RXD, and CAN_TXD). The system switches for digital input and output ports were used (SYS_SWDIO, SYS_SWCLK, and SYS_SW0). The ADC which shares with four external channels (ADC_IN0~ADC_IN3) was used to convert the selected analog inputs. The timer (PWM_OUT0) was used for pulse output generation. The internal reset (BOOT0, BOOT1, and nRESET) switches were used when the device operates in high temperature. If a system is detected for such an environment, the system automatically sends the signals back to the ports (BOOT0 and BOOT1). The external clock (HSE_IN and HSE_OUT) in the clock generator needs to be provided from ceramic oscillator (ABM8AIG-8.000MHZ-8-V1R-T, ABRACON, Spicewood, TX, USA) with two 10 pF ceramic capacitors because external clock can provide the designed accurate frequency to the system.

In Figure 4e, TXD and RXD are the transmission data input and reception data output of the CAN driver, respectively (CAN_TXD and CAN_RXD). The signals of high-level CAN bus line and low-level CAN bus line of the CAN communication driver (CANH and CANL) are the output to the battery MCU. The terminal resistance (R_term) was used to reduce reflection of the output voltages (CAN_H and CAN_L) for CAN communication output signal. The transient-voltage-suppression diode (D2) was used to protect the instantaneous overvoltage.

In Figure 4f, the LDO was used to provide a 3.3 V input voltage of the MCU from the BMS board. The several capacitors (0.1 and 10 µF) were used to stabilize the output voltage. The protection diode (D3, 1N4001) was used to block the circuit damage caused by reverse current when the power is off [48,49,50]. A light-emitting diode (LED, D1) was turned on through resistor (R9) when the port (DEBUG_LED) is low [51]. The SWD interface was used for weight and size reduction instead of the joint test action group (JTAG) [52]. The system switch and reset ports for digital input and output (SYS_SWCLK, SYS_SWDIO, SYS_SWO, and mRESET) in the SWD interfaces were connected to the header pin (CON3_PHO3) to check the message debugging. Serial UART communication was used to evaluate the device status. Therefore, the output generated from the boot loader ports (DEBUT_TX and DEBUG_RX) were also connected to the header pin (CON3_PHO1).

A real-time operating system kernel (FreeRTOS) was used to improve maintenance of the code reliability of the interrupt processing system. We implemented the hardware abstraction layer (HAL) driver after initializing the allocated vector table, operation clock division phase lock loop (PLL), and peripheral clock. The general-purpose input/output (GPIO) and initial value setting for the MCU peripheral, FreeRTOS porting, kernel start command were executed at set intervals. To provide a differential PWM output from the MCU, timer PWM generation 1 and 2 channels were used.

### 2.5. Battery Module Design

**Figure 5:**
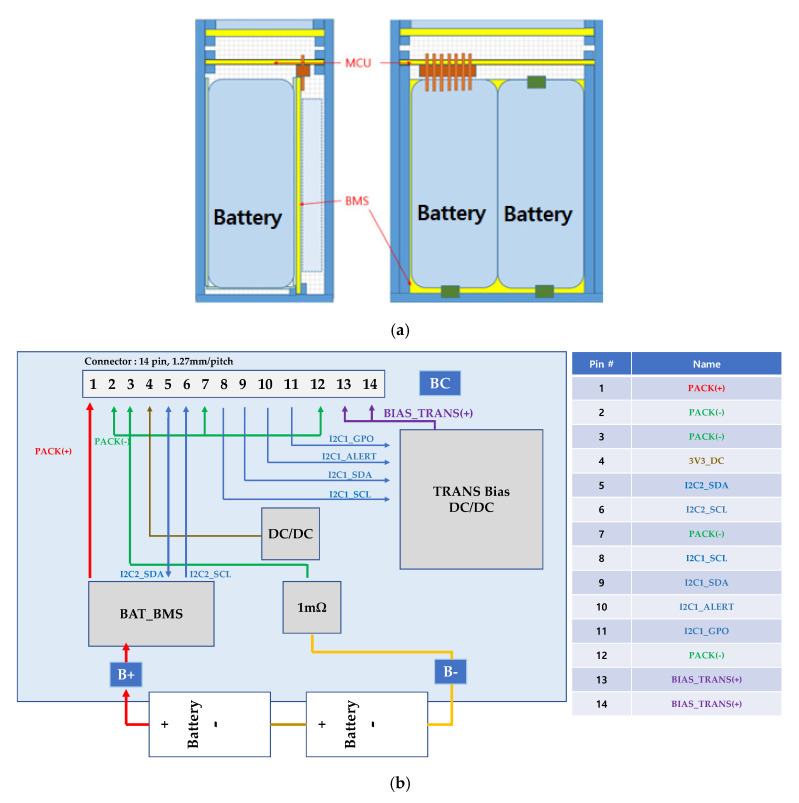

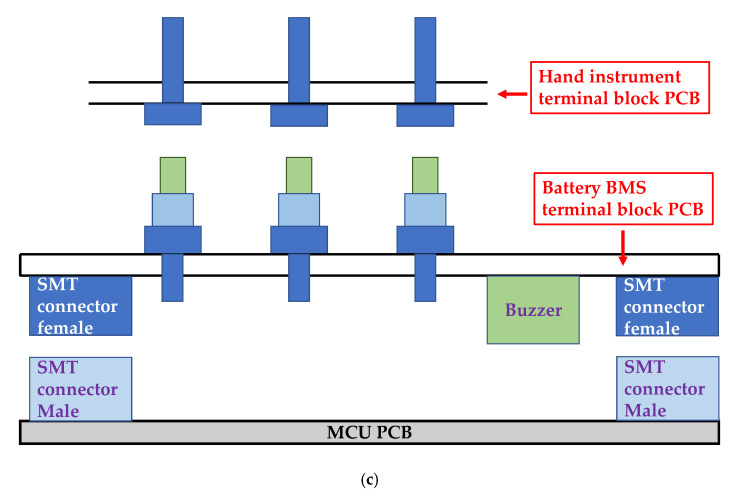
(**a**) BMS battery module configuration, (**b**) pulse code modulation (PCM)-DC/DC board configuration, and (**c**) terminal structure of the BMS and MCU.

For typical wireless ultrasound systems, the battery power is important for providing a constant voltage and current [42,53,54]. Thus, the upper part consists of the terminal block board for CAN communication and the power supply between the MCU board and the generator MCU board. The lower part consists of the BMS board with the battery cell, as shown in Figure 5a.

In Figure 5b, the MCU board controlling pulse code modulation (PCM)-DC/DC board connected with a 1.27 mm header 14 pin is shown, and it is controlled by system management Bus (SMBus) communication between the BAT_BMS IC and DC/DC IC with I2C1 and I2C2 interfaces. The battery management system was connected to first pin for positive battery voltage. This first pin is used to be charging and discharging electrodes. The 1 mΩ shunt resistor was used to measure the voltage in the second, third, seventh, and twelfth pins which are battery ground. A 3V DC through DC to DC converter (DC/DC) generated from LOD is the input of the MCU. The battery management system was connected to fifth and sixth pins for I2C2 interfaces for data communication and transmitting/receiving timing synchronization. The eighth, ninth, tenth, eleventh, and thirteenth and fourteenth pins were connected through DC to DC converter (TRANS Bias DC/DC) for serial data communication, data transmission, alert signal to the devices connected to SMBus, and GPIO signals, DC bias voltages for transformer, respectively.

As shown in Figure 5c, the buzzer is placed in the lower part of the BMS battery module terminal board printed circuit board (PCB) considering the component arrangement of the MCU PCB. The spring pogo pin of the through-hole type was used for the connection. For connecting/contacting the BMS battery module terminal block of the hand instrument terminal block, the mating pin of the pogo pin was used [55,56].

## 3. Results and Discussion

### 3.1. Ultrasound Transducer Characterization and Generator, MCU, and Battery Module Implementation

**Figure 6:**
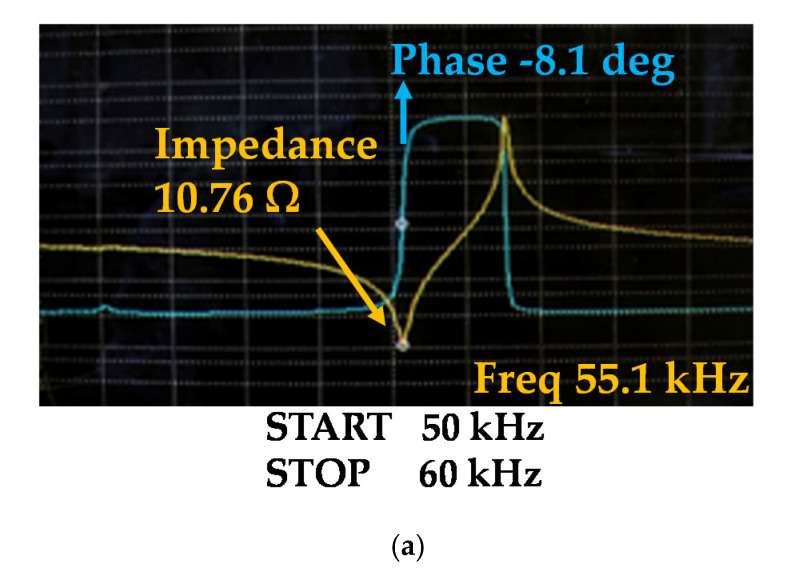

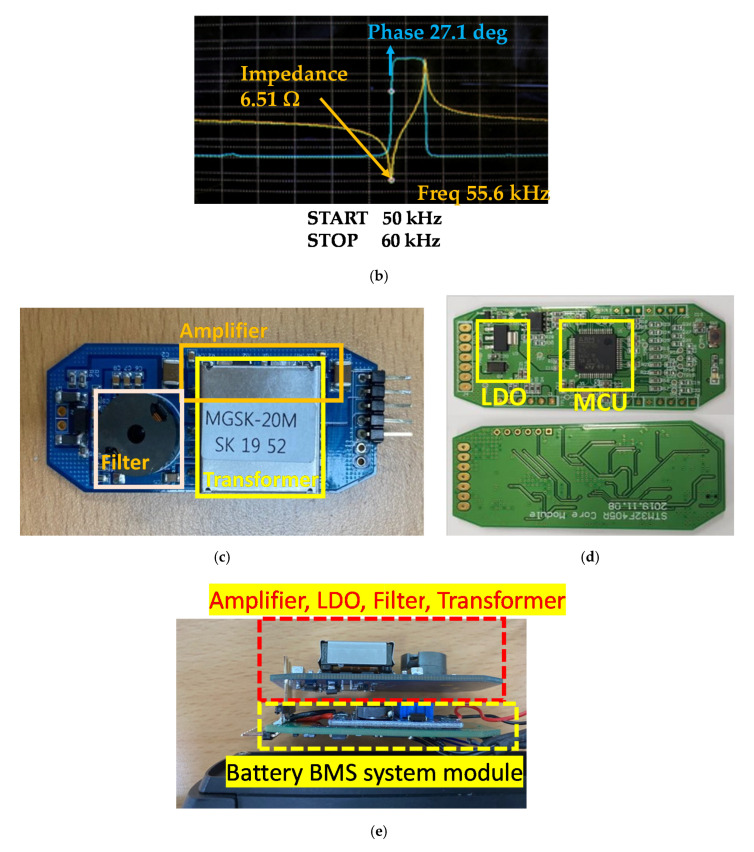
Measured impedance graphs (**a**) before the re-poling process and (**b**) after the re-poling process. (**c**) Frequency detector, transformer, level shifter, LDO, and amplifier, (**d**) MCU modules on the PCB, and (**e**) assembled components on the PCB.

Figure 6a,b show the measured impedance changes before and after the re-poling processes. The magnitude and frequency values of the electrical impedance at the resonance frequency before the re-poling process were 10.762 Ω and 55.125 kHz, respectively. After the re-poling process, the magnitude and frequency values of the electrical impedance at the resonance frequency changed to 6.51 Ω and 55.6 kHz, respectively.

Table 2 lists the measured UT characteristic values before and after the re-poling processes. The equation for *Q_m_* is shown below [57].

(4) $$Q_{m}=\frac{f_{a}^{2}}{2\pi f_{f}Z_{r}C\left(f_{a}^{2}-f_{r}^{2}\right)}$$

where, *f_r_* is the resonant frequency; *f_a_* is the anti-resonant frequency; *Z_r_* is the impedance at the resonant frequency, and *C* is the capacitance.

By comparing the before and after re-poling processes, sharp impedance characteristics are shown, where the calculated *Q_m_* value increased approximately 2 times to minimize heat generation because the bandwidth is related to power consumption [58,59].

Using the LCR meter, a UT fabricated based on the design specification showed approximately a 5.6 nF capacitance value. According to the capacitance result of the fabricated single crystal UT sample, there was a small error of approximately 1.7% in the modal analysis result. Therefore, the target frequency of 55.5 kHz ± 1% was reached. The measured impedance and resonant frequency are 55.6 kHz and 6.51 Ω, respectively, and the measured impedance and anti-resonant frequency are 56.6 kHz and 33.87 kΩ, respectively. When the re-poling process was applied, Δ*f*, which is the difference between the anti-resonance (*f_a_*) and resonant (*f_r_*) frequencies, was changed to a narrow band, and the impedance at the resonant frequency decreased, thus obtaining a high *Q_m_* [60,61]. The UT should have a high *Q_m_* to minimize the driving heat generation and enhance the power generation of the UT.

In Figure 6c, the transformer, filter, and amplifier are shown. Figure 6d shows the MCU module boards and LDO with the surface mount technology (SMT) components, and Figure 6e shows the assembled components on the PCB.

**Figure 7:**
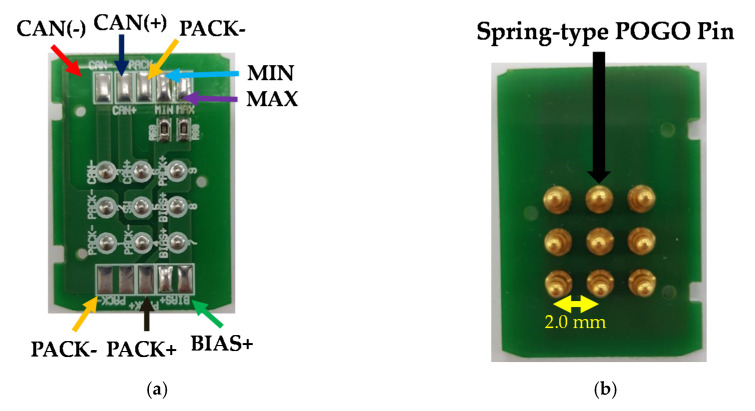

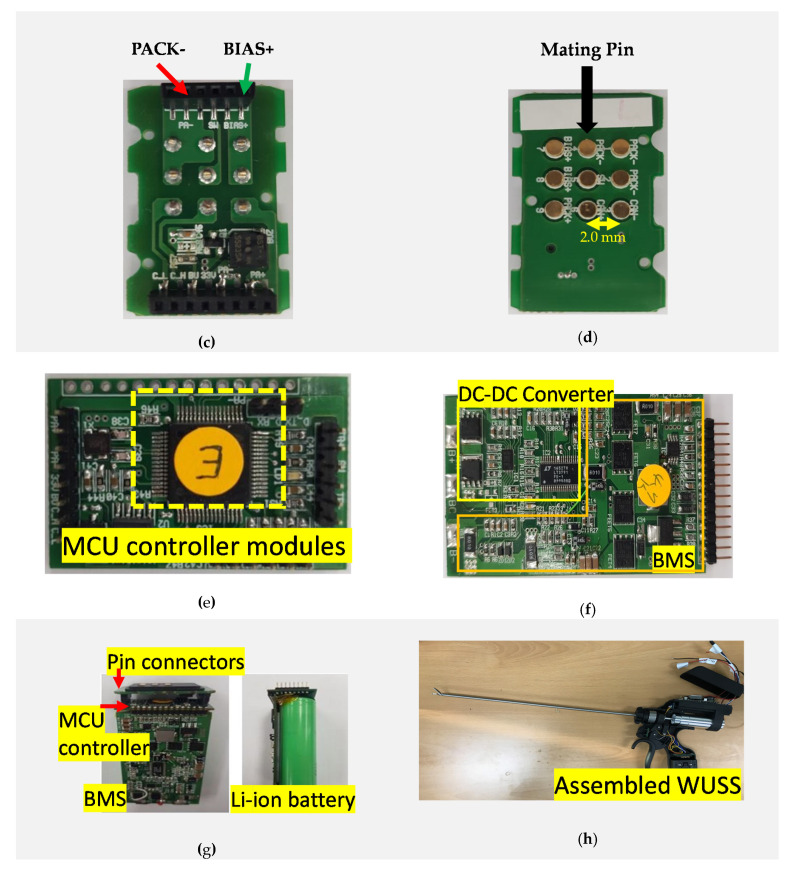
(**a**) Top and (**b**) bottom views of hand instrument pogo pinboards and (**c**) top and (**d**) bottom views of the mating pinboard. (**e**) MCU controller, (**f**) BMS/DC-DC converter boards, (**g**) Li-ion battery with control MCU boards, and (**h**) assembled WUSS.

As shown in Figure 7a,b, the pogo pin terminal board in the hand instrument was used for communication and the power supply between the generator and battery module for the WUSS. The spring-type pogo pin was selected to stabilize the maximum allowable current from the battery modules. The spring-type pogo pin which has less tension is useful to provide stable current such that it could be helpful to attach the battery management system terminal block accurately. For the switch input, the minimum/maximum values at the voltage divider circuit were determined by assigning one pin to check the minimum/maximum/open status using received ADC values. The buzzer (BST-5523SA, Bosan Hitech Co., Ltd., Incheon, Korea) was selected to obtain sufficient sound pressure (78 dBA) and power consumption (0.3 W) [62]. Figure 7c,d show the top and bottom views of the mating pinboard. The mating pinboard with 2.0 mm pitch pin connectors is a stack layer type.

Figure 7e,f show the MCU controller and DC-DC converter boards of the battery modules. The MCU controller in the battery module controls the switch input, CAN driver, PCM/BMS, buzzer. The PWM provides the voltage and current of the battery and performs the data status check through seamless communication with the generator MCU. The MCU communicated with the MCU by checking the battery overcharge/discharge protection circuits. The output current limit control can be changed to 10 A according to the hardware conditions. The BMS/DC-DC board checks the voltage supplied to the generator MCU and the bias voltages periodically according to the minimum/maximum switch input values and generates the outputs of the corresponding bias voltage level of the transformer according to the index value (0–30 level) requested from the generator MCU. Figure 7g,h show the Li-ion battery and battery with the MCU controller modules for the WUSS Li-ion battery, MCU control board, and pin connectors. In Figure 7g, the BMS board is interconnected by 2.00 mm pin connectors and pogo pinboards.

### 3.2. Performance Measurement of Wireless Ultrasound Surgical System

**Figure 8:**
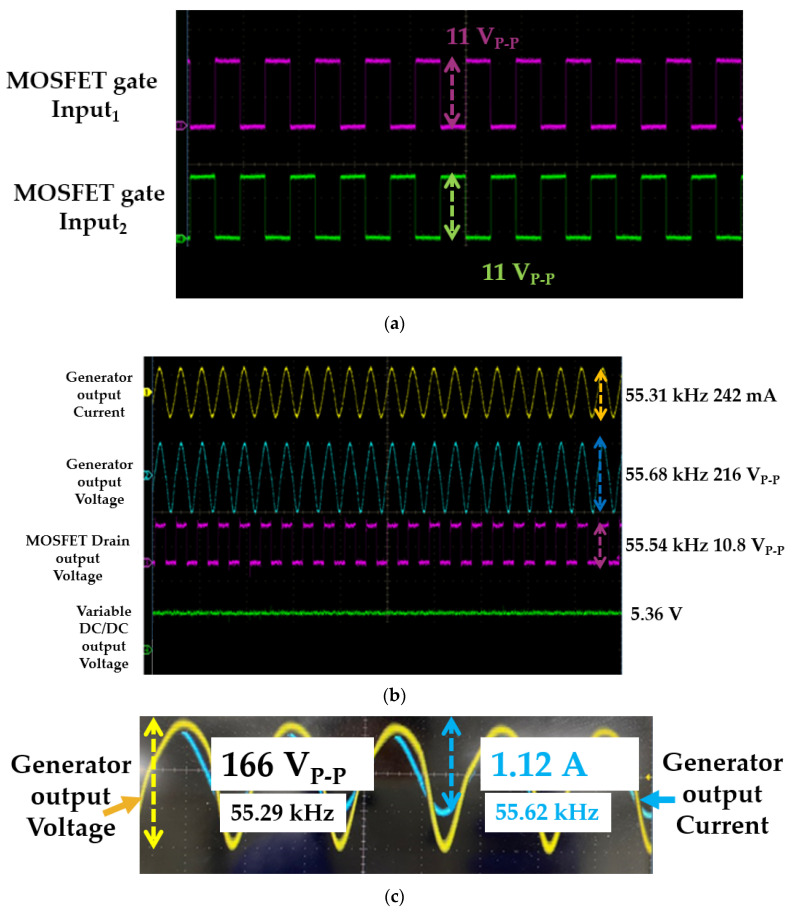
(**a**) Input waveforms of the metal-oxide-semiconductor field-effect transistor (MOSFET) driver, (**b**) output current and voltage amplitudes of the generator instrument before the matching circuit with the MOSFET driver drain output voltage and variable DC/DC output voltage, and (**c**) output current and voltage amplitudes of the generator instrument after the matching circuit.

Figure 8a shows the two gate input waveforms of the MOSFET driver, which are opposite phase angles. The amplitude of the input waveforms is approximately 11 V at the resonant frequency. Figure 8b shows the output current and voltage amplitudes of the generator instrument before the matching circuit with the MOSFET driver drain output voltage and variable DC/DC output voltages. The peak-to-peak voltage and current at the resonant frequency were 216 V and 242 mA, respectively. As shown in Figure 8b, the output voltage and current of the generator are not in the same phase. The maximum power of the transmitter-like generator can be delivered at the resonant frequency if the voltage and current of the generator are in phase [63,64,65]. After using matching circuits, the voltage and current of the generator in the same phase angles could be obtained to maximize the power transfer in the transmitter for the wireless system [66,67,68,69]. As shown in Figure 8c, the peak-to-peak output voltage and current amplitudes of the generator with the matching circuit were 166 V and 1.12 A, respectively, at the resonant frequency.

**Figure 9:**
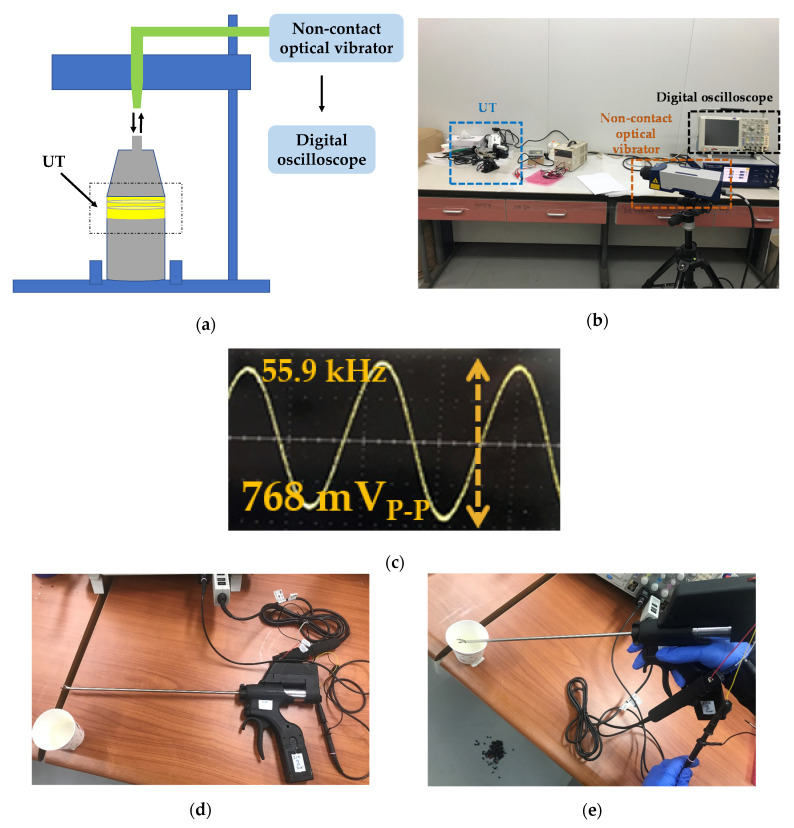
(**a**) Setup and (**b**) picture for the performance evaluation of the maximum amplitude and (**c**) measured maximum amplitude in the digital oscilloscope. (**d**) Setup for the performance evaluation of the applicable frequency ranges and (**e**) performance evaluation for power consumption reduction of the generator output.

Figure 9a,b show the setup of the maximum amplitude measurement of the UT using non-contact optical vibrator equipment (OFV-5000MD, Polytec GmbH Corp., Waldbronn, Germany) and a digital oscilloscope. As shown in Figure 9a,b, the laser sent from the non-contact optical vibration sensor was returned to the vibrator equipment to show the amplitude performances of the UT in the oscilloscope. The maximum amplitude of the WUSS was 96 µm, which is higher than that of a commercial system (80 µm). As shown in Figure 9c, the measured maximum frequency of the instrument is 55.9 kHz. The frequency and power consumption performances were measured for efficient WUSS performances.

To evaluate the usable frequency ranges (50 to 60 kHz), as shown in Figure 9d, the output frequencies of the generator were measured in the oscilloscope without the serial communication line and power supply cables. When the reference value of the applicable frequency was changed starting at 55 kHz with a 1 kHz step, the frequency values of the MCU PWM and generator matched between 50 and 60 Hz. As shown in Figure 9e, the power consumption at the maximum output was evaluated to verify the power consumption reduction rate when the generator output cable was connected by the current probe with water load. The power consumption of the developed WUSS was reduced by 3.6% compared to that of the current commercial WUSS. Table 3 shows the quantitative performance comparison between commercial and presented systems with respect to the parameters.

## 4. Conclusions

The WUSS should generate effective amplitude performances with efficient power consumption owing to limited battery modules since the amplitude and power consumption performances which are related with vibration amplitude are one of the most important parameters for WUSS hemostasis performance and efficient dissection time.

Current commercial WUSSs use Navy type I PZT-4 or Navy type III PZT-8 piezoelectric materials in the UT devices. To improve the performance of the UT devices for the WUSS, we used a piezoelectric material Mn: PIN-PMN-PZT (Mn-doped PIN-Pb(Mg_1/3_Nb_2/3_)O_3_-PbZrO_3_-PbTiO_3_) for the UT to demonstrate better electrical performances based on our best technical information. To design this UT, a newly developed re-poling process was also utilized to minimize the unwanted heat generation and achieve an improved quality factor of 2418 for the UT. The measured magnitude and frequency values at the resonance frequency of the electrical impedance are 6.51 Ω and 55.6 kHz, respectively, after the re-poling process. Therefore, the improved quality factor of the UT could be helpful to minimize the unwanted heat generation of the limited battery modules.

We also developed a custom-made generator, control MCU with an ARM controller, and battery management system modules to produce effective WUSS performances. The control MCU with an ARM controller and BMS battery modules were also designed to optimize the performance of the WUSS. Since the distorted signals of the UT could affect the WUSS performances with limited battery modules, we designed the transimpedance amplifier with 2nd harmonic termination circuit and transformer. In addition, the spring-type pogo pin with less tension was used to provide stable current from the battery module, thus improving the attachment durability to the battery management system. The peak-to-peak output voltage and current amplitudes of the generator with a matching circuit were measured as 166 V and 1.12 A, respectively, at the resonant frequency. Using the non-contact optical vibrator equipment, the maximum amplitude performance and the resonant frequency of the WUSS were 96 µm and 55.9 kHz, respectively. The power consumption of the developed WUSS was reduced by 3.6%, and the amplitude was improved by 20% compared to those of the current commercial system. Therefore, the developed WUSS could be beneficial for hemostatic performance during surgical operations owing to the reduced power consumption and improved output voltage amplitudes.

## Figures and Tables

**Table 1:** Metal material properties.

Parts	Material	Density (kg/m^3^)	Elastic Modulus (GPa)	Poisson’s Ratio	Longitudinal Velocity
Head horn	AL7075	2810	72	0.34	6358
Tail mass Tail bolt	Ti-6Al-4V	4420	111.09	0.33	6081

**Table 2:** Changed values before and after re-poling.

Parts	Before Re-Poling	After Re-Poling	Difference
*f_r_* (Hz)	55,125	55,600	575
*f_a_* (Hz)	56,550	56,600	−50
*Z_r_* (Ω)	10.762	6.51	−3.84
*C* (Farad)	5.6 × 10^−9^	5.6 × 10^−9^	0
*Q_m_*	1231.19	2418	1187.68

**Table 3:** Quantitative performance comparison between commercial and presented systems.

Parameters	Commercial System	Presented System
Vibration amplitude	80 µm	96 µm
Output voltage amplitude	138.3 V	166 V
Power consumption	14.5 W	14 W
